# Characterisation of the natural environment: quantitative indicators across Europe

**DOI:** 10.1186/s12942-017-0090-z

**Published:** 2017-04-26

**Authors:** Graham Smith, Marta Cirach, Wim Swart, Audrius Dėdelė, Christopher Gidlow, Elise van Kempen, Hanneke Kruize, Regina Gražulevičienė, Mark J. Nieuwenhuijsen

**Affiliations:** 1 Staffordshire University, Leek Road, Stoke-on-Trent, ST4 2DF UK; 2 ISGlobal, Barcelona Institute for Global Health, Barcelona, Spain; 3 Universitat Pompeu Fabra (UPF), Barcelona, Spain; 4 CIBER Epidemiología y Salud Pública (CIBERESP), Barcelona, Spain; 5 Centre for Sustainability, Environment and Health, National Institute for Public Health and the Environment (RIVM), PO Box 1, 3720 BA Bilthoven, The Netherlands; 6 Department of Environmental Science, Vytauto Didžiojo Universitetas, K. Donelaičio g. 58, 44248 Kaunas, Lithuania

**Keywords:** Health, Nature, Parks, Urban planning, Physical activity, Social interaction, Stress

## Abstract

**Background:**

The World Health Organization recognises the importance of natural environments for human health. Evidence for natural environment-health associations comes largely from single countries or regions, with varied approaches to measuring natural environment exposure. We present a standardised approach to measuring neighbourhood natural environment exposure in cities in different regions of Europe.

**Methods:**

The Positive Health Effects of the Natural Outdoor environment in TYPical populations of different regions in Europe (PHENOTYPE) study aimed to explore the mechanisms linking natural environment exposure and health in four European cities (Barcelona, Spain; Doetinchem, the Netherlands; Kaunas, Lithuania; and Stoke-on-Trent, UK). Common GIS protocols were used to develop a hierarchy of natural environment measures, from simple measures (e.g., NDVI, Urban Atlas) using Europe-wide data sources, to detailed measures derived from local data that were specific to mechanisms thought to underpin natural environment-health associations (physical activity, social interaction, stress reduction/restoration). Indicators were created around residential addresses for a range of straight line and network buffers (100 m–1 km).

**Results:**

For simple indicators derived from Europe-wide data, we observed differences between cities, which varied with different indicators (e.g., Kaunas and Doetinchem had equal highest mean NDVI within 100 m buffer, but mean distance to nearest natural environment in Kaunas was more twice that in Doetinchem). Mean distance to nearest natural environment for all cities suggested that most participants lived close to some kind of natural environments (64 ± 58–363 ± 281 m; mean 180 ± 204 m). The detailed classification highlighted marked between-city differences in terms of prominent types of natural environment. Indicators specific to mechanisms derived from this classification also captured more variation than the simple indicators. Distance to nearest and count indicators showed clear differences between cities, and those specific to the mechanisms showed within-city differences for Barcelona and Doetinchem.

**Conclusions:**

This paper demonstrates the feasibility and challenges of creating comparable GIS-derived natural environment exposure indicators across diverse European cities. Mechanism-specific indicators showed within- and between-city variability that supports their utility for ecological studies, which could inform more specific policy recommendations than the traditional proxies for natural environment access.

**Electronic supplementary material:**

The online version of this article (doi:10.1186/s12942-017-0090-z) contains supplementary material, which is available to authorized users.

## Background

The positive public health effects of access to natural environments have been widely demonstrated [1]. Increased physical activity and social contacts, psychological restoration, stress reduction, and a reduction in pollutants such as noise pollution, air pollution and heat, have been proposed as possible mechanisms that underpin these associations [1, 2].

Inconsistency and variation in indicators used to characterise exposure to green or natural space have often made it difficult to compare results from different studies [3–5]. This limits the identification of associations that can inform public health and urban planning policy. As noted elsewhere [3, 6], there are several sources of variation. First, scale of reported measures can range from land parcels (statistical/administrative units; e.g., [7–9]) to entire cities (e.g., [10]). Second, neighbourhood definitions or buffer sizes vary, from those thought to equate to a 5-min walk (300–500 m) or 10-min walk (800–1000 m) [11], to larger distances [12]. Third, buffers can also be Euclidean (straight-line) or network (using street/pathway networks); a selection that should be governed by the nature of the specific inquiry [13]. Fourth, the GIS procedures to assign environments to buffers can vary (e.g., containing features, intersecting features). Finally, the specific metrics reported can include number, area or density of spaces per unit of land area, or use distance to nearest environment [3].

The definitions of natural environment used in previous studies is another potential limitation, particularly when exploring specific mechanisms. Studies have often used a broad definition of green or natural environment, and lacked the quantification of environments by type or quality. Measures of the density and proximity to natural environments are derived from existing, often coarse, cartographical databases of land use [such as the English Generalised Land Use Database (GLUD)] or classifications of remotely sensed land cover data, such as the European CORINE data, which maps areas of at least 25 ha down to a resolution of 100 m. The level of resolution should be appropriate for the purpose of the study. For example, in PHENOTYPE, to study a diverse range of natural environment across different cities, it was important to be as inclusive as possible and use a resolution that could capture the smaller natural environments that can be beneficial for health. A key data source used in previous research is Landsat imagery, which has 30 m resolution and is deemed sufficient for most applications [14].

Normalised Difference Vegetation Index (NDVI), derived from satellite imagery, is widely used as a single measure of neighbourhood greenness. NDVI has shown associations with health outcomes with relative consistency [9, 15]. It is appropriate when considering mechanisms linking natural environments and health, which do not rely on use of/visits to the space (stress reduction, attention restoration, mitigation of environment pollutants) [16]. When considering specific behaviours or uses of natural environments, however, greater specificity in the exposure measures is required.

Some recent studies have evaluated the use of European data to create a consistent green space indicator; for example, using Urban Atlas, which can have resolution up to 100 times than CORINE (another European source) [17]. Others have proposed a methodology for standardising the estimation of park accessibility [18]. Such studies provide potentially useful tools for planners and policy makers in terms of providing consistent measures. But the granularity of classification for specific health-related mechanism assessment might still be lacking. For example, the type of environment indicator required for an assessment of the associations between natural environment exposure and physical activity could be expected to be different to indicators required to assess associations with psychological restoration.

Positive Health Effects of the Natural Outdoor environment in TYPical populations in different regions in Europe (PHENOTYPE) is a collaborative European research project. Its overarching aim was to produce a more robust and comparable evidence base on links between exposure to natural outdoor environment and human health and well-being for north western, eastern and southern Europe. In particular, PHENOTYPE was concerned with the investigation of the proposed underlying mechanisms of stress reduction/restorative function, physical activity, social interaction, mitigation of exposure to environmental hazards. This was the first study designed to examine these mechanisms simultaneously in a large sample (N = 4000 participants) in different European cities using the same methodology [5].

This paper has a number of aims:

1. To develop a common classification of natural environments across cities in different regions of Europe comprised of a hierarchy of indicators from simple measures, such as NDVI that are easily obtained for all the study areas, to detailed measures created for specific mechanism assessment.
2. To explore between-city variation in key measures of natural environment exposure and examine the relationship between the simple and detailed measures.
3. To discuss lessons learned and next steps for examining natural environment in relation to health and the associated mechanisms of stress reduction/restorative function, physical activity and social interaction, across multiple countries.

## Methods

PHENOTYPE aimed to apply a common research design to study a broad range of natural environment exposures and using comparable objective measures of natural environments. This included detailed assessment of the natural environment in four cities (Barcelona, Spain; Doetinchem, the Netherlands; Kaunas, Lithuania; and Stoke-on-Trent, UK) that offered diversity in terms of size and composition, amount and type of natural environment, and represented different regions of Europe. The overall PHENOTYPE study design has been detailed elsewhere [5]. The methods reported here provide further detail on the neighbourhood sampling, the approach to characterising the natural environment and the creation of mechanism-specific indicators of natural environment.

### Neighbourhood and participant sampling

Within each city, a two-stage sampling design was adopted. Firstly, neighbourhoods were purposefully selected to maximize within-city environmental and socioeconomic variation. Second, a random sample of adults were recruited from selected neighbourhoods and natural environments around their residential addresses were characterised using the GIS methods reported in this paper.

**Fig. 1:**
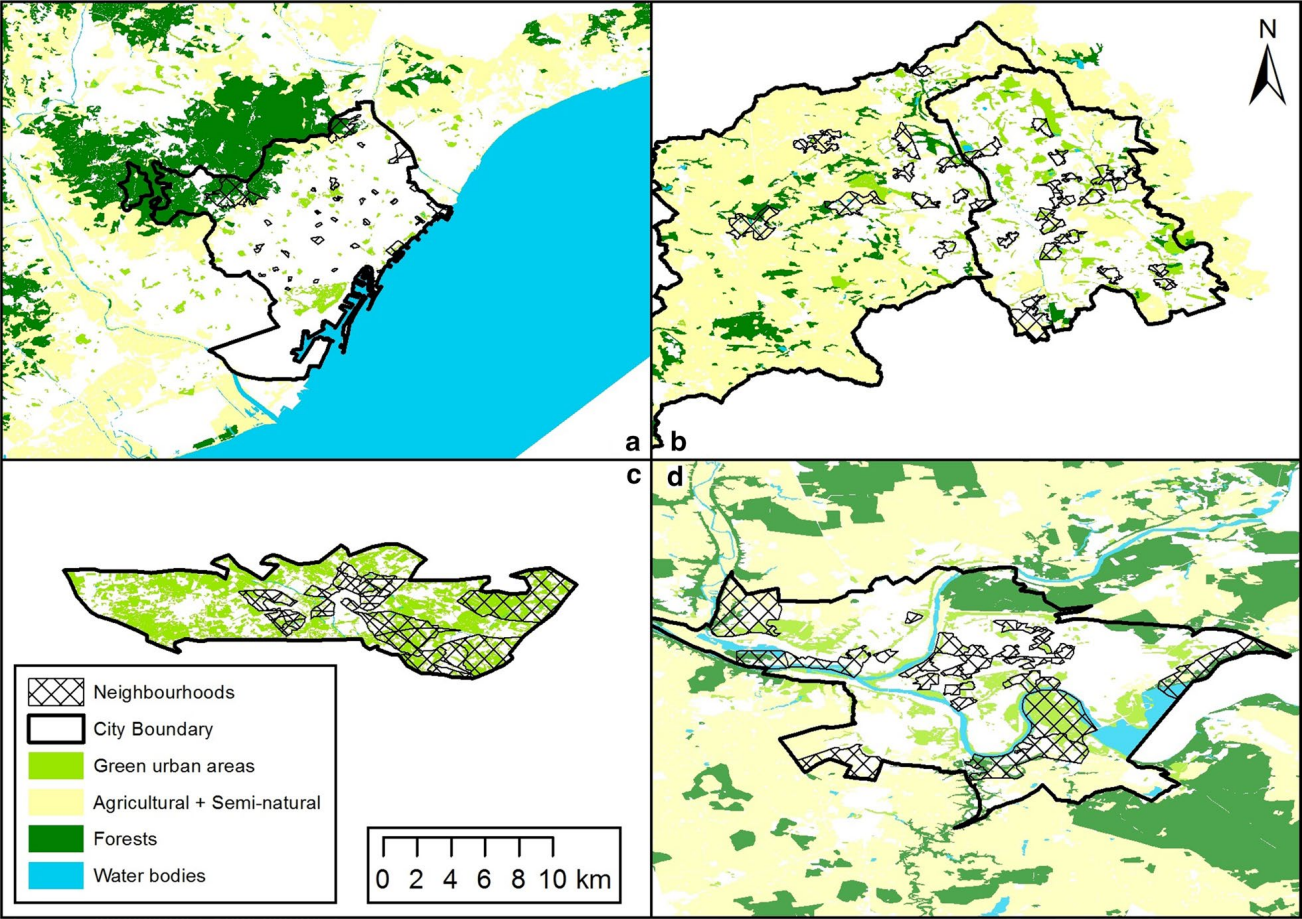
A map of each of the four cities that are included in the study, showing selected neighbourhoods and the location of natural environments. **a** Barcelona, **b** Stoke-on-Trent, **c** Doetinchem and **d** Kaunas (this map contains natural environments identified using EEA Urban Atlas data for all cities except Doetinchem (which shows natural environments derived from ‘Top10 NL’ at a comparable scale). All maps are presented at the same scale, 1:450,000)

Existing statistical or administrative units were used to define neighbourhoods within each city (Table 1). The aim was to use spatial units that were as similar as possible in terms of population size. The spatial units of selected neighbourhoods in Barcelona were much smaller in physical size than the other cities and the population density was much higher. Doetinchem was the smallest city, both in size and population (Table 1). All neighbourhoods were ranked by socio-economic status (SES) and access to natural environment. A total of 30 areas with sufficient adult population were selected within each city and placed into tertiles of natural environment and quintiles of SES. Two neighbourhoods from each combination of SES tertiles and natural environment quintiles were selected (2 × 3 × 5). For access to natural environments, Urban Atlas, which has a smallest spatial unit of 2500 m^2^ for urban green space, was used for Stoke-on-Trent, Barcelona and Kaunas. Since Urban Atlas was not available for Doetinchem, an extra step was undertaken before categorisation. Based on comparisons of green space data from another database (‘Top10 nl’) in another Dutch city for which Urban Atlas was available (Utrecht), similar categories were defined and used to extract natural environments larger than 2500 m^2^ (Fig. 1). Those categories showed 82% comparability with the Urban Atlas categories. For SES no comparable data existed for the four cities. Therefore, partners used their own local data (local deprivation index in Barcelona and Stoke-on-Trent, household income in Doetinchem and education levels in Kaunas).

**Table 1:** Summary of spatial units used for neighbourhood selection in each city

Study area	Stoke-on-Trent	Barcelona	Kaunas	Doetinchem
Study area population	363,421 (year 2010)	1,631,259 (year 2011)	319,213 (year 2011)	56,247 (year 2012)
Adult population	214,194 (aged 16–59)	1,169,445 (aged 20–74)	227,578 (aged 19–65)	33,491 (aged 20–65)
Study area size (km^2^)	304.41	102.16	155.98	79.64
Population density (per km^2^)	1193.85	15,967.69	2046.50	706.27

Spatial unit	LSOA	Census areas	Voting districts	Neighbourhoods
Count of spatial units	241	1061	116	83
Mean population	1508	1537.5	3400	670
Min. population	1024	466	684	0
Max.population	4200	7291	6767	4410
SD	264	419.4	780	830
Spatial unit size (km^2^)				
Mean size	1.26	0.11	1.34	0.96
Min size	0.13	0.011	0.09	0.09
Max size	42.90	14.28	8.58	7.36
SD	4.22	0.64	1.85	1.22
Spatial unit pop density (per km^2^)				
Mean density	3360.73	42,832.30	8695.04	1986.42
Min density	35.26	79.46	211.19	6.00
Max density	10,302.4	140,572.26	34,371.59	8068.00
SD	1996.52	22,856.28	7932.57	2156.86
Local natural environment data				
Data source(s)	Stoke-on-Trent City Council Green Space Audit 2012 OS MasterMap Topographic layer	Mapa Ecològic de Barcelona (3a edició) Topogràfic updated 2011	Inventory of green areas of Kaunas city (Kaunas city administration)—applied to Urban Atlas boundaries—as at 2012	Top10Vector (state mapping agency) 2006
Resolution or smallest unit	1:1250	1:800, 1:5000	2500 m^2^	1:5000

### Natural environment characterisation

A number of different types of exposure indicators were produced for each participant: Normalized Difference Vegetation Index (NDVI); number of natural spaces within a straight-line or network distance from the participant’s home; total amount (total area) of natural spaces within a straight-line or network distance from the participant’s home; and distance to nearest accessible natural space (or type of spaces). These types of indicators were produced for two levels of classification:

- *Simple indicators* Derived from broadly categorised (green or greenness, blue), freely available, Europe-wide, consistent data, such as Urban Atlas and NDVI derived from LandSat satellite images.
- *Detailed indicators* Derived from detailed spatial and categorical data from local sources in each city with a common classification of natural environments applied to green and blue spaces.

The majority of the indicators used some form of distance or buffer calculation to define an ‘area of exposure’ or ‘area of assessment’. The distances used were not necessarily the same for each indicator, but were dependent on the mechanism and type of space; e.g., neighbourhood greenness using NDVI was calculated using small buffer sizes (100–500 m), whereas recreation spaces were measured within larger buffers, such as 500 and 1000 m as people can travel to access spaces for specific recreational purposes.

Both Euclidean (straight-line) buffers and network buffers were used to define an area or exposure; the latter used a modified road network to calculate distance from a participant’s home address to the nearest environment or any given environment. As noted elsewhere [13], the choice of Euclidean versus network buffers can potentially have a considerable effect of the estimated exposure and context should be considered. For PHENOTYPE, we used both based on the need to accommodate the study of multiple mechanisms thought to link natural environment exposure and health. For example, to gain health benefit from physical activity or social activity in the natural environments requires that individuals visit the space. Therefore, network buffers are appropriate. For the psychological processes of stress reduction or restoration that can be facilitated through viewing natural environments, straight-line buffers can be used.

There is no evidence-based cut-off for the maximum distance associated with health benefits [14]. A number of recurring distances were used in the creation of the PHENOTYPE indicators:

- *100* *m* This has been used elsewhere to capture immediate neighbourhood surrounding greenness (e.g., [19]).
- *300* *m* A commonly used threshold for accessibility [13] that was central to the Accessible Natural Greenspace Standards (ANGSt) developed by Natural England [20], and the recent World Health Organisation standard [17].
- *500 and 1000* *m* Widely used approximations of 5- and 10-min walking distances, respectively, often used when considering network distances in physical activity and walking studies [11].

### Simple indicators

#### NDVI

NDVI is an indicator of green vegetation density based on the difference between visible red and near-infrared surface reflectance. It is used to represent the level of vegetation or greenness with a given location, on a scale of −1 to +1 (where higher values indicate higher vegetation density). Mean NDVI values within straight-line buffer distances (commonly used for NDVI [14]), of 100, 300 and 500 m were calculated as estimates of neighbourhood surrounding greenness. The source data were Landsat data (USGS), using Landsat 8 satellite images at 30 m × 30 m spatial resolution. We aimed to find cloud-free images within the greenest season (May–September) in 2011–2013, the relevant period for this study.

#### Urban atlas

The aim of the Urban Atlas Indicators was to develop standardised indicators of the amount of natural space accessible within a range of distances of each participant addresses using existing standardised European data. Urban Atlas provides reliable, comparable, high-resolution land use maps for 305 Large Urban Zones and their surroundings (more than 100,000 inhabitants as defined by the Urban Audit) for the reference year 2006 [21]. The following land use categories (and codes) were used to extract natural environments: Green Urban Areas (14,100), Agricultural and Semi Natural Areas (20,000), Forests (30,000), Water bodies (50,000).

Accessible (or walkable) natural environments were included if they intersected (overlapped) a participant network buffer. The entire area of a space was included even if it only partially overlaps the buffer. The count and total area of intersecting natural spaces were calculated for all participants and all network buffer distances.

### Classification of natural environments and detailed indicators

To produce a comparable classification of natural environments across study areas, local data were collated on natural environments, and current definitions and categories were identified that could be used to characterise natural spaces/environments in each study area. A common classification of environments was then applied to the local data. This was initially based on a combination of planning guidance documents used in the UK [22, 23]. This classification and the definitions used were compared to those in the other cities. The classifications are based on the primary purpose of the space. PAN 65 was used as the basis for the classification because it has been adapted to create a 1:1250 scale green space map for Scotland (http://greenspacescotland.org.uk/scotlands-greenspace-map.aspx). Many of the natural environment categories were present in the classifications already used in individual cities. Categories were matched and adapted across cities to create the final classification.

The classification, definitions and criteria used to create groupings of natural environment types for specific mechanism assessment are summarised in Table 2. The inclusion criteria for each subset can be summarised as:

**Table 2:** Natural environment classification and typology definitions and subsets for analysis of specific health-related mechanisms

Level 1	Level 1.1	Level 2	Type(s) of space included	Mechanism group (and size criteria)		
				Stress reduction/restoration (all types)	Physical activity	Social contact
Green	Urban green space	Parks	Urban parks	Y (≥0.25 ha)	Y (≥0.5 ha)	Y (≥0.25 ha)
		Semi-natural/natural	Biodiversity areas, conservation areas, nature reserves, protected areas, heritage sites?	Y (≥0.25 ha)	Y (≥0.5 ha)	Y (≥0.25 ha)
		Formal recreation	Playgrounds and sports fields (not within parks)	Y (≥0.25 ha)	Y (≥0.25 ha)	Y (≥0.25 ha)
		Civic space	Squares, gardens,	Y (any size)	Y (≥0.5 ha)	Y (any size)
		Functional/amenity	Allotment, cemetery, amenity spaces, Institutional (school, hospital grounds etc.)	Y (≥0.25 ha)	Y (≥0.5 ha)	Y (≥0.25 ha)
		Natural/green corridor	Traffic free/natural: pathways, trails and cycle paths	Y (≥0.25 ha)	Y (≥0.25 ha)	Y (≥0.25 ha)
		Derelict/vacant		N	N	N
		Residential gardens	Private gardens	N	N	N
		Other natural features	Street greenery	Y (≥0.25 ha)	N	N
	Woodland/forests	Woodland/forests	Woodland/forests	Y (≥0.25 ha)	Y (≥0.5 ha)	Y (≥0.25 ha)
	Rural and agricultural land	Rural and agricultural land	Rural and agricultural land	Y (≥0.25 ha)	Y (≥0.5 ha)	Y (≥0.25 ha)
	Country parks	Country parks	Country parks	Y (≥0.25 ha)	Y (≥0.5 ha)	Y (≥0.25 ha)
Water	Freshwater (inland water)	Lakes/reservoirs/ponds (standing water bodies)		Y (≥0.25 ha)	Y (≥0.5 ha)	Y (≥0.25 ha)
		Rivers, streams, canals (linear water features)		Y (≥0.25 ha)	Y (≥0.25 ha)	Y (≥0.25 ha)
	Marine/coastal	Including beeches (type of coastline)		Y (≥0.25 ha)	Y (≥0.25 ha)	Y (≥0.25 ha)

*Stress reduction and restoration* All natural environments were included apart from derelict urban green space, which is assumed not to provide a ‘pleasant’ environment for people to access or to view. This inclusive approach was based on the rationale that stress reducing and restorative benefits may be conferred from simply viewing natural environments [24], and are not necessarily specific to environments of a certain size, accessibility or type.

*Physical activity* All natural environments that were publically accessible, and/or provided dedicated physical activity opportunities (e.g., playground or sports field) and were large enough to support some level of physical activity (≥0.5 hectares) were included. This latter criterion was within the range used in other physical activity studies (e.g., 0.4 ha [25], 0.8 ha [26, 27] and deemed appropriate for the study areas).

*Social interaction/cohesion* All natural environments that were publically accessible were included. This is based on the rationale that social interaction occurs in the natural environment, which must, therefore, be accessible, but is not necessarily limited by size or type.

*Exposure to environmental hazards* All natural spaces were considered important regardless of size and accessibly. There are insufficient data to match spaces to particular types of environmental hazard that they mitigate.

## Results

### Simple indicators

**Fig. 2:**
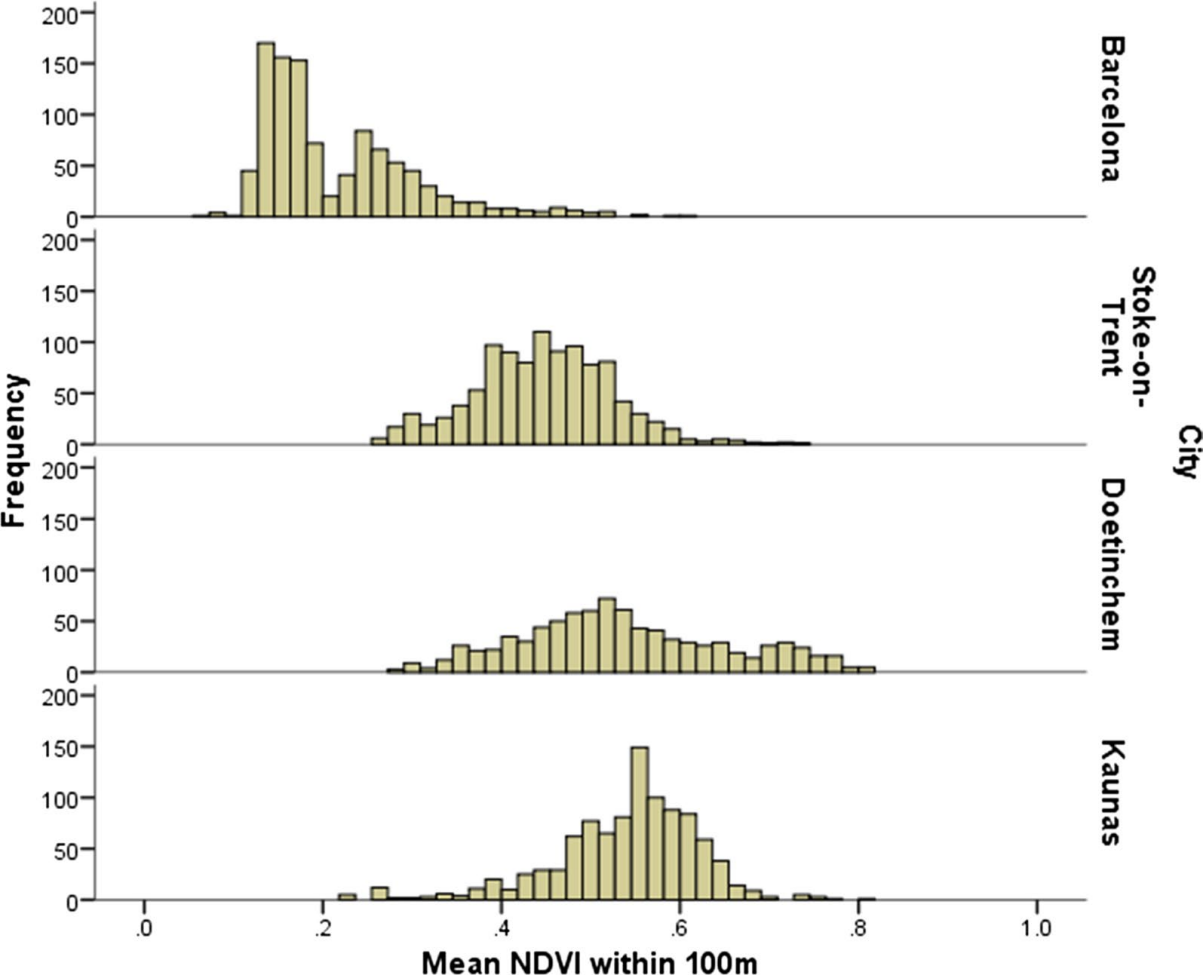
Histogram showing distribution of mean NDVI within 100 m by city

Indicators created using NDVI and Urban Atlas are presented in Table 3 (and Additional file 1: Table S1) showing the descriptive statistics across the whole study and by city. Figure 2 reports the mean NDVI within 100 m (and Additional file 1: Figure S1 shows NVDI within 300 m by city). There was a high correlation between the 100 m indicator and both the 300 and 500 m indicators (r = 0.94 and 0.91 respectively; data not shown), but Doetinchem had the highest mean level of greenness (NDVI) at 0.55 for 300 m. There was a similar distribution of the mean NDVI across participants from Doetinchem, Kaunas and Stoke-on-Trent. Barcelona, however, had a much lower mean NDVI at 0.22, and an uneven distribution of values. This different distribution highlighted the difference between participant exposures in urban and sub-urban neighbourhoods; the majority of Barcelona residents lived in urban areas with little residential greenness, with a small number living in greener sub-urban neighbourhoods.

**Table 3:** Basic indicators of natural environment exposure using LandSat and Urban Atlas

City	All		Barcelona		Stoke-on-Trent		Doetinchem		Kaunas	
n	n = 3946		n = 1044		n = 1044		n = 861		n = 997	
	Mean	SD	Mean	SD	Mean	SD	Mean	SD	Mean	SD
NDVI measures										
Mean NDVI within 100 m	0.43	0.16	0.22	0.09	0.45	0.08	0.54	0.12	0.54	0.08
Mean NDVI within 300 m	0.44	0.16	0.22	0.10	0.47	0.08	0.55	0.09	0.54	0.07
Mean NDVI within 500 m	0.45	0.16	0.23	0.10	0.48	0.08	0.56	0.08	0.54	0.07
Urban Atlas measures										
Straight-line distance to										
Nearest natural space (m)	180.05	203.85	362.65	280.81	106.29	75.48	64.00	58.22	166.28	143.67
Nearest green space (m)	183.62	206.18	362.71	280.76	110.36	78.25	67.93	59.67	172.69	159.37
Nearest blue space (m)	1321.29	1258.08	2564.30	1635.64	973.60	723.95	509.19	354.32	1085.07	661.87
Street-network buffer										
Green spaces within 300 m (n)	1.38	1.57	0.73	1.16	1.33	1.20	2.75	2.00	0.92	1.07
Count of green spaces within 500 m (n)	3.01	2.60	1.91	2.29	2.99	1.85	5.62	2.76	1.95	1.66
Count of green spaces within 1000 m (n)	9.64	6.58	7.68	7.38	9.12	3.54	16.83	5.73	6.03	3.30
Total area of green spaces within 300 m (ha)	13.24	33.10	9.92	35.54	19.67	45.32	13.98	20.95	9.35	20.42
Total area of green spaces within 500 m (ha)	27.14	54.17	15.57	41.82	46.12	74.76	27.87	30.05	18.75	50.70
Total area of green spaces within 1000 m (ha)	81.35	131.70	37.58	73.22	129.75	154.21	86.83	46.57	71.78	178.12
Count of blue spaces within 300 m (n)	0.07	0.30	0.00	0.00	0.12	0.42	0.16	0.40	0.01	0.13
Count of blue spaces within 500 m (n)	0.18	0.54	0.05	0.21	0.29	0.72	0.37	0.70	0.04	0.23
Count of blue spaces within 1000 m (n)	0.58	1.11	0.22	0.53	0.83	1.52	1.12	1.23	0.23	0.54

For Urban Atlas (Table 3), there was some between-city variation in distance to the nearest natural environment (green and blue), but the relatively low mean values for all cities indicated close proximity of most residents to natural environments. The count indicators derived from Urban Atlas network buffers showed some different between-city patterns (compared with distance to nearest). For example, Barcelona and Kaunas had approximately similar mean numbers of green spaces within 300, 500 and 1000 m. For total count of green space, Doetinchem still appeared to have the best access to natural environments, whereas mean values for total area of green space indicated that Stoke-on-Trent had the best access for all the different network buffers. Data in Additional file 1 highlight that mean area values are skewed by very large green spaces (with the exception of Doetinchem, the smallest city).

### Classification

Table 4 shows the total area (and percentage) of Level 1 and Level 2 natural environment types (defined in Table 5) by city. These data highlight the different make-up of each city in terms of the prominent types of natural space. Marked differences were seen for semi-natural areas, which comprised very little of the area for Barcelona, compared with other cities. In Stoke-on-Trent, there was a considerable proportion of natural environment classified as formal recreation spaces (which are distinct from parks), but not elsewhere. Civic spaces in Kaunas comprised a particularly high proportion of the city, although this is likely to include some areas that could be classified as amenity spaces in other cities. Such examples of possible ‘misclassification’ did not pose a threat to between-city comparability of indicators derived from these environment classifications as they were subsequently grouped for each mechanism (rather than exploring only specific types; see “Methods” section).

**Table 4:** Natural environments mapped and classified in each city (hectares and proportion of area)

Level 1	Level 1.1	Level 2	Total area^a^							
			Barcelona		Stoke-on-Trent		Doetinchem		Kaunas	
			ha	%	ha	%	ha	%	ha	%
Green	Urban green space	Parks	657.11	61.68	527.25	11.65	71.01	16.71	743.76	15.57
		Semi-natural/natural	3.23	0.30	1347.70	29.77	34.80	8.19	509.10	10.66
		Formal recreation	1.38	0.13	729.06	16.10	3.21	0.76	0.00	0.00
		Civic space	291.96	27.41	7.80	0.17	9.23	2.17	3332.22	69.75
		Functional/amenity	34.00	3.19	1111.07	24.54	100.42	23.64	1.34	0.03
		Natural/green corridor	73.58	6.91	176.68	3.90	57.66	13.57	54.26	1.14
		Derelict/vacant	4.04	0.38	627.82	13.87	4.37	1.03	136.49	2.86
		Residential gardens								
		Street greenery					135.10	31.80		
		Total urban green space	1065.30		4527.37		424.87		4777.16	
	Non-urban green space	Woodland/forests	0.00	0.00	1861.99	10.38	859.55	16.25	1344.66	100.00
		Rural and agricultural land	0.00	0.00	16,077.11	89.62	4430.39	83.75	0.00	0.00
		Country parks	1696.31	100.00	0.00	0.00	0.00	0.00	0.00	0.00
		Total non-urban green space	1696.31		17,939.10		5289.94		1344.66	
Water	Freshwater (inland water)	Lakes/reservoirs/ponds (standing water bodies)	0.00	0.00	97.38	78.58	20.02	30.91	631.10	47.88
		Rivers, streams, canals (linear water features)	9.00	27.17	26.55	21.42	44.74	69.09	687.08	52.12
	Marine/coastal	including beeches (type of coastline)	24.12	72.80	0.00	0.00	0.00	0.00	0.00	0.00
	Total blue space		33.13		123.93		64.76		1318.17	1318.17
Total area of natural environments			2794.75		22,590.40		5779.57		7440.00	7440.00

^a^% Figures are calculated as proportion of total area for each of the Level 1.1 group

**Table 5:** Natural environment classification and typology definitions and subsets for analysis of specific health-related mechanisms

Level 1	Level 1.1	Level 2 (typology)	Type(s) of urban green space included	Definition (purpose of space)
Green	Urban green space	Parks	Urban parks	Areas of land normally enclosed, designed, constructed, managed and maintained as a public park. Accessible, high quality opportunities for informal recreation and community events. These will include a variety of features which may include formal footpaths, play space, sports areas, trees, planted beds and ponds
		Semi-natural/natural	Biodiversity areas, conservation areas, nature reserves, protected areas, heritage sites?	Wildlife conservation, biodiversity and environmental education or awareness. Areas of undeveloped or previously developed land within or adjoining an urban area with residual natural habitats or which have been planted or colonised by vegetation and wildlife
		Formal recreation	Playgrounds and sports fields (not within parks)	Playgrounds: Areas providing safe and accessible opportunities for children’s play, such as fixed play equipment, adventure play and skate parks. Sports: Large and generally flat areas of grassland or specially designed surfaces, used primarily for designated sports i.e. playing fields, tennis courts, bowling greens. This class includes natural and artificial playing surfaces
		Civic space	Squares, gardens,	Squares, streets, predominantly of hard landscaping that provide a focus for pedestrian activity and make connections for people and for wildlife, where trees and planting are included. Gardens, Areas of land normally enclosed, designed, constructed, managed and maintained as a garden. These will include a variety of features which may include formal footpaths, trees, planted beds and ponds. They are small in size and function in a similar way to public squares
		Functional/amenity	Allotment, cemetery, amenity spaces, institutional (school, hospital grounds etc.)	School: land normally enclosed and associated with a school. Amenity: Unenclosed greenspace surrounding high-rise flats and other residential buildings. Enclosed land around other public institutions (hospitals, police stations, fire stations, universities, colleges, nursing homes). Unenclosed, landscaped areas providing visual amenity or separating different buildings or land uses for environmental, visual or safety reasons, i.e. road verges or greenspace in business parks, and used for a variety of informal or social activities such as sunbathing, picnics or kickabouts. Enclosed land associated with churches and other places of worship. Land used currently or previously as a place of burial and land associated with crematoriums
		Natural/green corridor	Traffic free/natural: Pathways, Trails and cycle paths	Linear routes linking different areas within a town or city as part of a designated and managed network and used for walking, cycling or horse riding, or linking towns and cities to their surrounding countryside or country parks. These may link greenspaces together. Accessible greenspace, such as that associated with disused railway lines and paths
		Derelict/vacant		Disused natural areas with no clear purpose (‘stalled spaces’)
		Residential gardens	Private gardens	Enclosed individual or shared gardens associated with residential properties
		Street greenery	Street greenery	This class should be used for open space associated with road and rail which provide visual amenity/landscaping only, i.e. they would not be used by people for recreation

Level 1	Level 1.1	Level 2 (typology)	Definition (purpose of space)
Green	Non-urban green space	Woodland/forests	Areas of land normally enclosed, designed, constructed, managed and maintained as a woodland. These areas are dominated by tree cover but can include a variety of features which may include formal footpaths, and visitor facilities
		Rural and agricultural land	Enclosed lowland agricultural land. Unenclosed upland agricultural land
		Country parks	Many are located near or within towns and cities and therefore close to where people live. All provide a wide range of opportunities for recreation
Water	Freshwater (inland water)	Lakes/reservoirs/ponds (standing water bodies)	Areas of open water and associated green or hard landscaping/waterfront space
		Rivers, streams, canals (linear water features)	Canal towpaths, accessible river corridors and the associated greenspace
	Marine/coastal	Including beeches (type of coastline)	Waterfront promenades, predominantly of hard landscaping that provide a focus for pedestrian activity

### Detailed indicators (derived from classification)

**Fig. 3:**
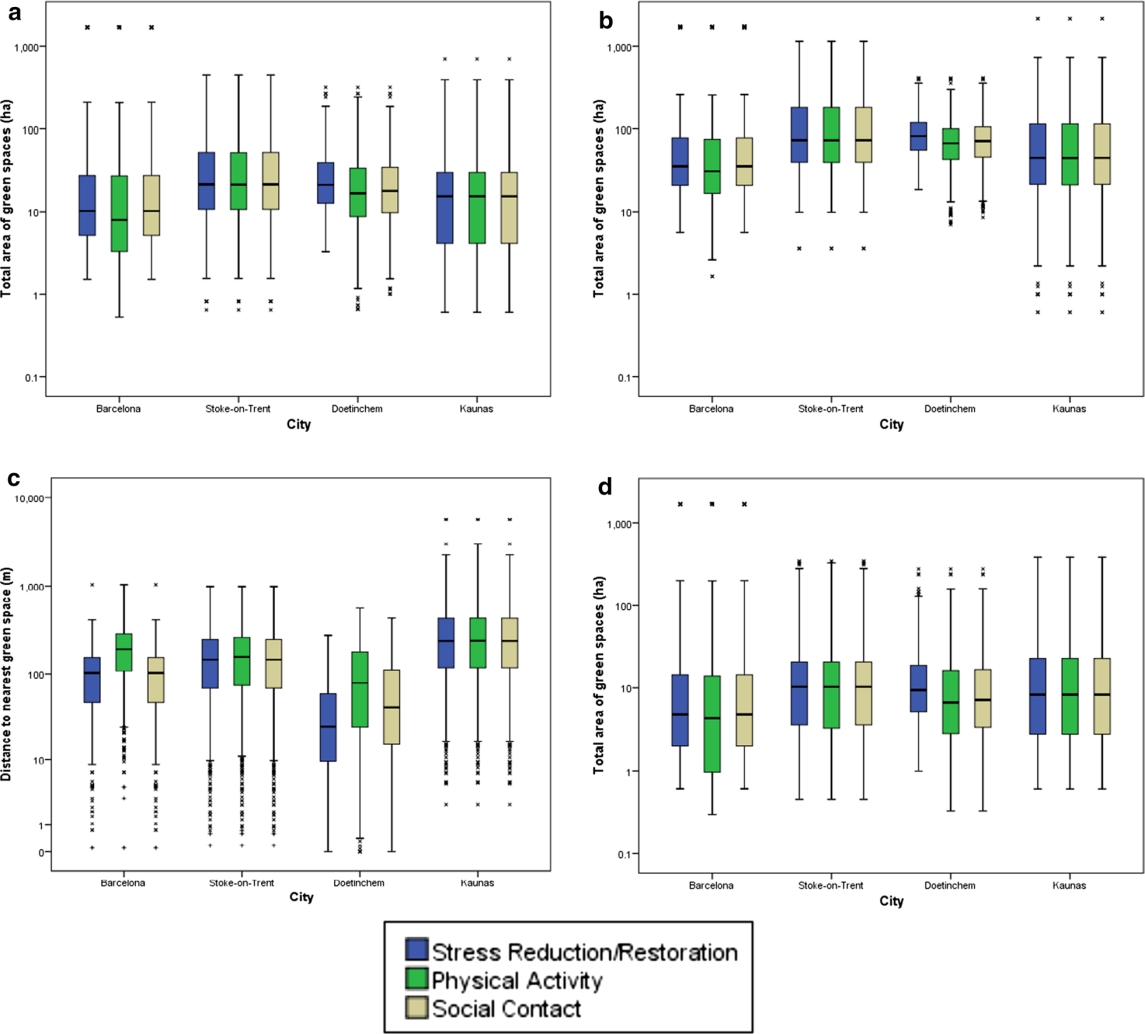
Green space exposure area measures for mechanism assessment by city (presented with logarithmic scale). **a** Distance to nearest (m), **b** total area within 300 m, **c** total area within 500 m and **d** total area within 1000 m

Figures 2 and 3 show indicators derived from detailed local data, by city, based on each mechanism. The distance to nearest data showed that the mechanism subsets for physical activity and social interaction captured more variation in Doetinchem and Barcelona (compared with the stress/restoration mechanism subset, which included all spaces), particularly for the physical activity mechanism (Fig. 3a). For total area within 300 m, a similar pattern was observed, which was still observable at 500 and 1000 m, albeit less marked (Fig. 3b–d).

**Fig. 4:**
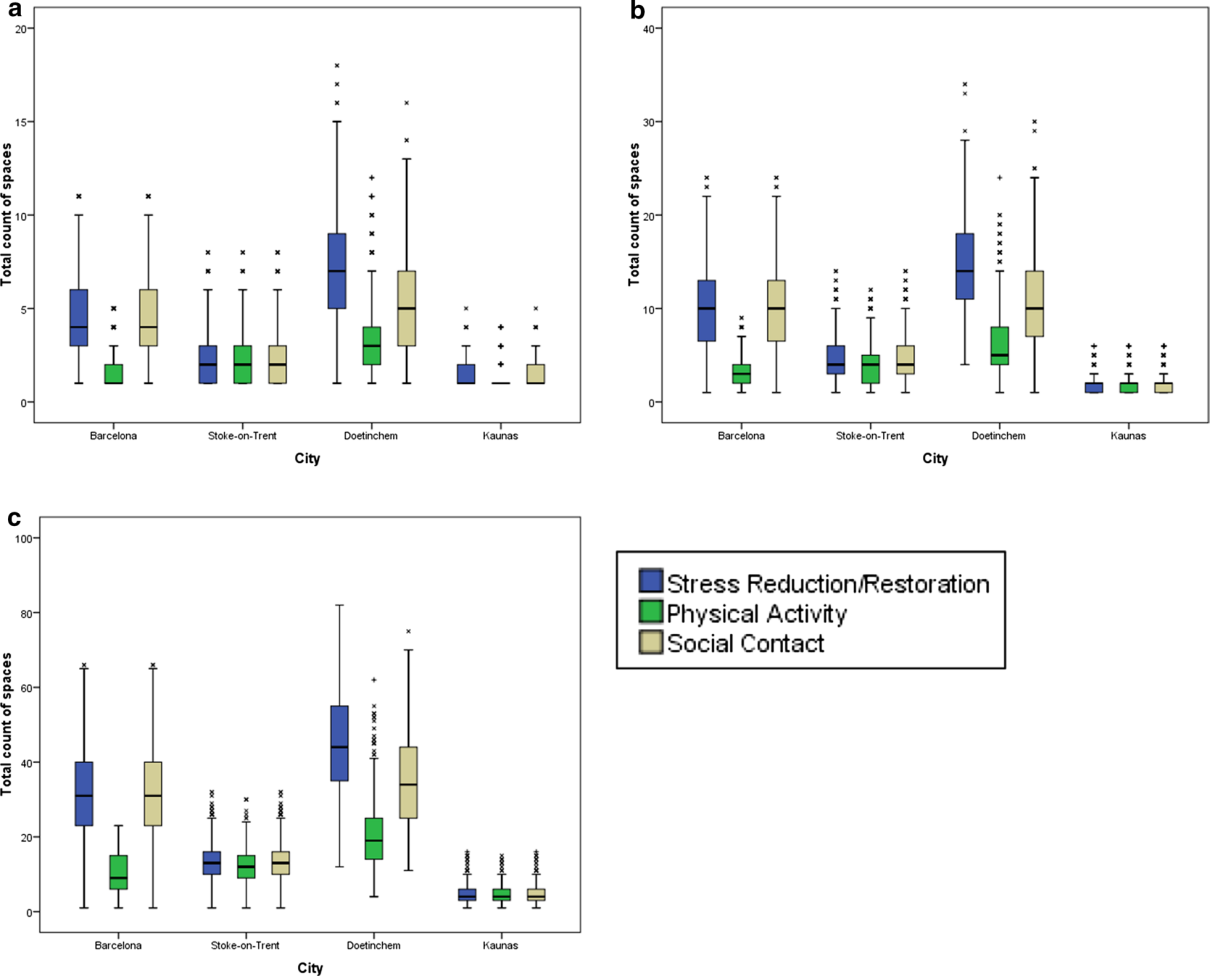
Green space exposure count for mechanism assessment by city (presented with logarithmic scale). **a** Count of spaces within 300 m, **b** count of spaces within 500 m and **c** count of spaces within 1000 m

Figure 4 shows the distribution of green space counts, by city, for each network buffer (300, 500, 1000 m). Again, in Barcelona and Doetinchem, there were clear differences between indicator subsets for mechanisms, particularly physical activity, which were not seen for Stoke-on-Trent or Kaunas.

**Fig. 5:**
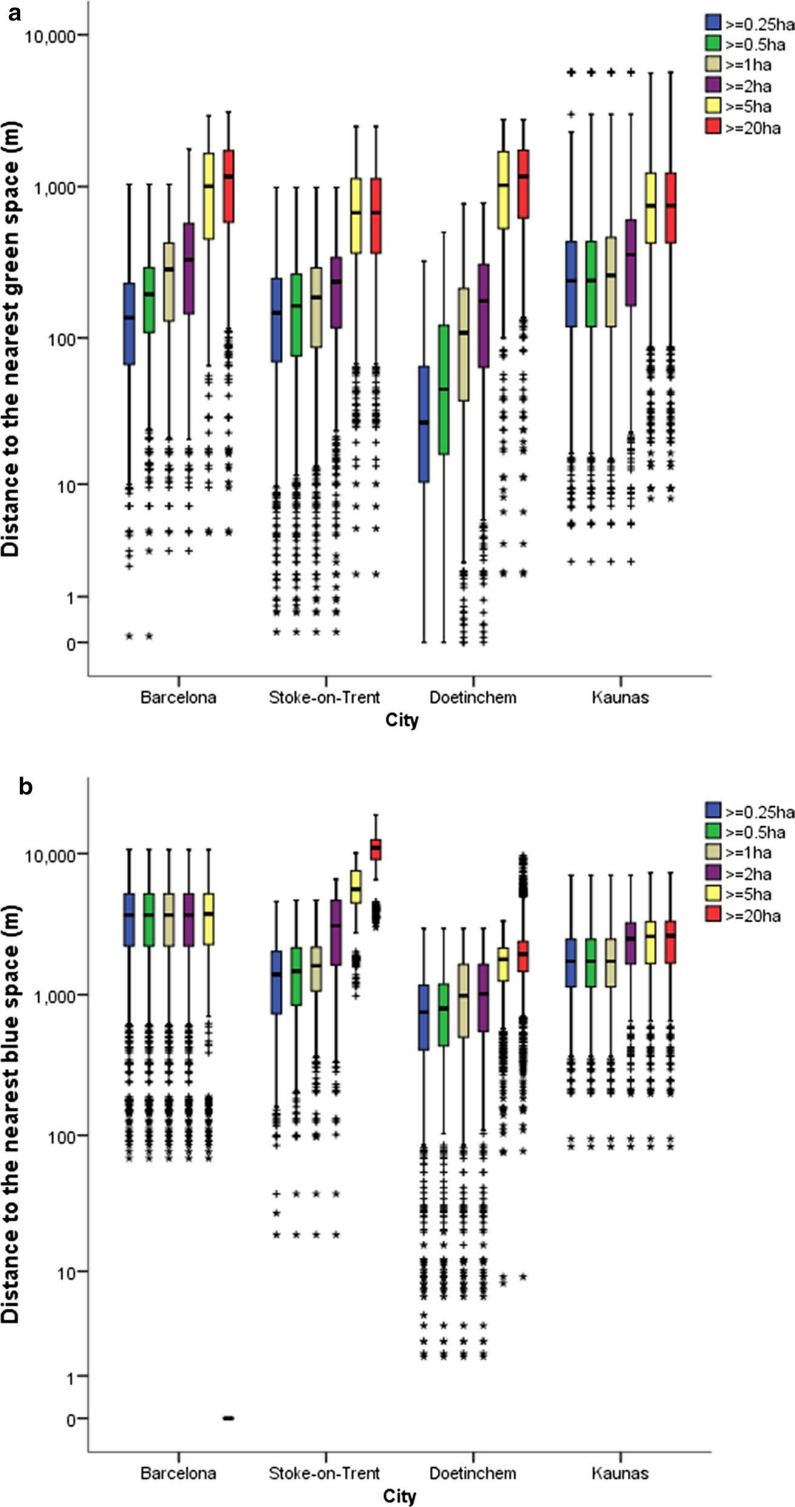
**a** Distance to nearest green space by cumulative size category for each city (presented with logarithmic scale) and **b** distance to nearest blue space by cumulative size category for each city (presented with logarithmic scale)

Figure 5a and b show the distribution of values for the network distance to nearest green space and blue space, respectively, by size category for each city. These show difference in access to larger spaces. Figure 5a also highlights the very close proximity to smaller spaces (<1 ha) within Doetinchem compared with other cities. When looking at access to green spaces ≥5 ha Doetinchem had the worst access, but values were similar across all cities.

## Discussion

This paper demonstrates the feasibility of creating comparable GIS-derived variables that characterise the natural environment features from urban and suburban regions of four diverse cities in different regions of Europe. It also presents a method of generating indicators specific to the mechanisms thought to link natural environment and health. A key rationale for PHENOTYPE was that international studies should maximise variability of environments to avoid underestimating associations between natural environment exposure and physical activity, stress reduction/psychological restoration and social contacts. Using the common approach to the detailed characterisation of environment reported here, the results showed within- and between-city variability in natural environment exposures, which suggested that these indicators should be suitable for ecological studies.

As reported elsewhere, widely available data/natural environment indicators, such as NDVI and Urban Atlas, are useful for identifying high level, broad associations in health-related studies. They are appealing because they are available consistently across countries/cities and can, therefore, be useful for wider policy purposes. However, there are issues [28] that limit their usefulness when trying to unpick the mechanisms underpinning the natural environment-health association. For example, in neighbourhoods that have generally high levels of green and natural environments (such as Doetinchem in the present paper), broad classifications of green and blue environments may not capture sufficient variation for use in ecological studies. Also, the coarseness of some data create issues for derived indicators. For example, in Urban Atlas, indicators based on count measures might not be entirely accurate as adjacent spaces might appear as separate spaces in the data, but exist as a single space. For total area-based indicators, there are limitations associated with edge effects; i.e., those living near to very large spaces can appear as outliers with extremely high levels of access, particularly for those on the edges of towns/cities with neighbouring rural land.

This is the first classification developed with a focus on indicators specific to the mechanisms posited to underpin natural environment-health associations. Often, studies have used indicators that include all urban green space (e.g., [29, 30]), considered only exposure to blue space [31, 32], or have limited to single types; often focusing on parks in physical activity studies [33, 34]. Single type classifications can be problematic for studies across diverse cities, regions and countries as some types of space may not be present in some areas, but an equivalent function could be served by a different class or type of environment. Our approach aimed to produce exposure measures to further current understanding of the complex natural environment-health association through being inclusive in relation to the ‘types’ of environment (from amenity spaces to formal recreation or coastal areas), whilst being sensitive to some of the attributes that are necessary to support different activities that are relevant to different mechanisms.

A linear distance from the home of 300 m has been proposed as a standard for green space accessibility measures [17]. Our data show that this could be more useful if combined with a type-based classification of green space to capture variability. For example, we observed differences in total area and count of green spaces within a 300 m network derived from all green space compared versus green spaces that are supportive of physical activity (≥0.5 ha, publically accessible).

### Limitations

As expected for an international study making use of secondary GIS data, there are limitations in the ways data can be collected and integrated. The aim was to make best use of existing data to measure the natural environments in a detailed manner that was appropriate for mechanism assessment, overcoming common issues associated with inconsistencies in neighbourhood selection, GIS-related methods and characterisation of environments.

There are a number of limitations relating to the simple indicators that have been discussed earlier in this section and ultimately confirm our rationale for creating the detailed, mechanism-specific measures. Limitations relating to these detailed indicators include, firstly, the lack of exposure variation in Doetinchem. Despite the stratified approach to neighbourhood sampling, the high level of greenness in the city is likely to have reduced our ability to detect differences. It is still important to look at differences using the mechanism-specific indicators in areas with large amounts of overall green space as the relationships between environment and health related mechanisms, such as physical activity, could be misrepresented. Second, there were issues in creating the common classification across cities because of cultural differences. For example, as discussed above, certain types of space exist in some countries and not others (e.g., ‘squares’ in Barcelona). Although this can be overcome, to do so requires an understanding of the cultural context to identify the equivalent environments or environments that serve an equivalent function in other countries. This is necessarily on a case-by-case basis and becomes more problematic as the number of cities or countries and the cultural diversity increases. Third, none of the existing data sources included a measure of quality. Quality measurement of natural environment is complex, increasingly so when trying to quality assess a broad range of green and blue environments across diverse cities. For PHENOTYPE, bespoke streetscape and natural environment quality audit tools were developed [5], but the absence and/or lack of consistency in quality scores from existing environment data sources remains a limitation. In recent years, there has been interest in user-generated or participatory approaches, such as ‘Wikification of GIS by the masses’ [35] or ‘Voluntary Geographical Information’ (VGI) [36], and Public Participation GIS (PPGIS) [37]. Such approaches have the potential to crowd source data from large numbers of people, which could provide good coverage [38], but issues around data quality and consistency would need to be explored given the complexity and subjectivity of natural environment quality assessment [37].

## Conclusions and future research

This paper demonstrates both the feasibility and challenges of creating comparable GIS-derived natural environment exposure indicators across diverse European cities. The detailed quantitative indicators presented here will be combined with quality audit data and detailed participant data from the computer assisted personal interviews (on health, use and perceptions of natural environment, physical activity, social interaction, etc.) to improve current understanding of the link between natural environment and health beyond that possible using simple quantity measures. These could be further developed using more recent data sources that allow greater specificity in natural environment characterisation across Europe (e.g., OpenStreetMap). Our results can potentially lead to more informed practices and policies in health, city planning, and parks and recreation sectors, and this method of measuring resident natural environment exposure could be a first step to creating measures to be used by planners and policy makers. Planners could use such measures to assess towns and cities on mechanism specific ‘supportiveness’; i.e., the extent to which the combination of natural environment within a given area support physical activity, mental health and other co-benefits. The result would be more specific policy recommendations for natural environment provision than using simple proxies for access (e.g., X m^2^ of green space per head of population or within a distance of X m of residential dwellings).

## Additional file

**Additional file 1.** Supplementary table and figures.
