# Supplementary material for: Characterisation of the natural environment: quantitative indicators across Europe
Source: Int J Health Geogr. 2017 Apr 26;16:16. doi: 10.1186/s12942-017-0090-z (PMC5406880; doi:10.1186/s12942-017-0090-z)
Supplement: Supplementary file 1 — Additional file 1. Supplementary table and figures. [file 12942_2017_90_MOESM1_ESM.docx]

Table S1. Basic indicators of natural environment exposure using LandSat and Urban Atlas

| City | All | | | Barcelona | | | Stoke-on-Trent | | | Doetinchem | | | Kaunas | | |
| --- | --- | --- | --- | --- | --- | --- | --- | --- | --- | --- | --- | --- | --- | --- | --- |
| n | n = 3946 | | | n = 1044 | | | n = 1044 | | | n = 861 | | | n = 997 | | |
|  | 25% | 50% | 75% | 25% | 50% | 75% | 25% | 50% | 75% | 25% | 50% | 75% | 25% | 50% | 75% |
| ***NDVI measures:*** |  |  |  |  |  |  |  |  |  |  |  |  |  |  |  |
| Mean NDVI within 100m | 0.3080 | 0.4603 | 0.5475 | 0.1546 | 0.1812 | 0.2658 | 0.3954 | 0.4483 | 0.4973 | 0.4577 | 0.5224 | 0.6111 | 0.4980 | 0.5563 | 0.5919 |
| Mean NDVI within 300m | 0.3286 | 0.4847 | 0.5519 | 0.1603 | 0.1917 | 0.2529 | 0.4187 | 0.4745 | 0.5209 | 0.4849 | 0.5354 | 0.6026 | 0.4936 | 0.5423 | 0.5906 |
| Mean NDVI within 500m | 0.3261 | 0.4937 | 0.5584 | 0.1739 | 0.1968 | 0.2412 | 0.4244 | 0.4757 | 0.5324 | 0.5102 | 0.5480 | 0.6063 | 0.4920 | 0.5439 | 0.5859 |
| ***Urban Atlas measures:*** |  |  |  |  |  |  |  |  |  |  |  |  |  |  |  |
| *Straight-line distance to:* |  |  |  |  |  |  |  |  |  |  |  |  |  |  |  |
| Nearest natural space (m) | 50.96 | 110.91 | 232.36 | 150.95 | 300.83 | 486.11 | 46.62 | 88.85 | 150.51 | 17.78 | 45.47 | 101.87 | 65.90 | 113.90 | 233.72 |
| Nearest green space (m) | 53.74 | 112.82 | 237.81 | 151.12 | 300.83 | 486.11 | 50.33 | 92.97 | 156.02 | 19.88 | 50.38 | 109.69 | 65.90 | 114.25 | 243.10 |
| Nearest blue space (m) | 451.53 | 902.13 | 1712.90 | 1253.55 | 2562.59 | 3721.19 | 405.83 | 780.91 | 1455.84 | 223.78 | 469.14 | 719.50 | 567.03 | 959.56 | 1485.48 |
| *Street-network buffer:* |  |  |  |  |  |  |  |  |  |  |  |  |  |  |  |
| Green spaces within 300m (n) | 0.00 | 1.00 | 2.00 | 0.00 | 0.00 | 1.00 | 0.00 | 1.00 | 2.00 | 1.00 | 2.00 | 4.00 | 0.00 | 1.00 | 1.00 |
| Count of green spaces within 500m (n) | 1.00 | 3.00 | 4.00 | 0.00 | 1.00 | 3.00 | 2.00 | 3.00 | 4.00 | 4.00 | 5.00 | 7.00 | 1.00 | 2.00 | 3.00 |
| Count of green spaces within 1000m (n) | 5.00 | 8.00 | 13.00 | 3.00 | 5.00 | 10.75 | 7.00 | 9.00 | 11.00 | 13.00 | 16.00 | 20.00 | 3.00 | 6.00 | 8.00 |
| Total area of green spaces within 300m (ha) | 0.00 | 2.34 | 10.88 | 0.00 | 0.00 | 2.67 | 0.00 | 4.11 | 14.80 | 2.51 | 5.55 | 13.90 | 0.00 | 1.74 | 6.82 |
| Total area of green spaces within 500m (ha) | 2.47 | 9.97 | 28.29 | 0.00 | 2.19 | 9.97 | 5.02 | 16.80 | 39.48 | 8.23 | 15.33 | 37.09 | 1.81 | 6.29 | 22.23 |
| Total area of green spaces within 1000m (ha) | 18.11 | 42.99 | 100.12 | 5.48 | 14.98 | 37.89 | 28.21 | 62.77 | 162.85 | 42.98 | 83.31 | 120.69 | 17.75 | 34.71 | 81.21 |
| Count of blue spaces within 300m (n) | 0.00 | 0.00 | 0.00 | 0.00 | 0.00 | 0.00 | 0.00 | 0.00 | 0.00 | 0.00 | 0.00 | 0.00 | 0.00 | 0.00 | 0.00 |
| Count of blue spaces within 500m (n) | 0.00 | 0.00 | 0.00 | 0.00 | 0.00 | 0.00 | 0.00 | 0.00 | 0.00 | 0.00 | 0.00 | 1.00 | 0.00 | 0.00 | 0.00 |
| Count of blue spaces within 1000m (n) | 0.00 | 0.00 | 1.00 | 0.00 | 0.00 | 0.00 | 0.00 | 0.00 | 1.00 | 0.00 | 1.00 | 1.50 | 0.00 | 0.00 | 0.00 |


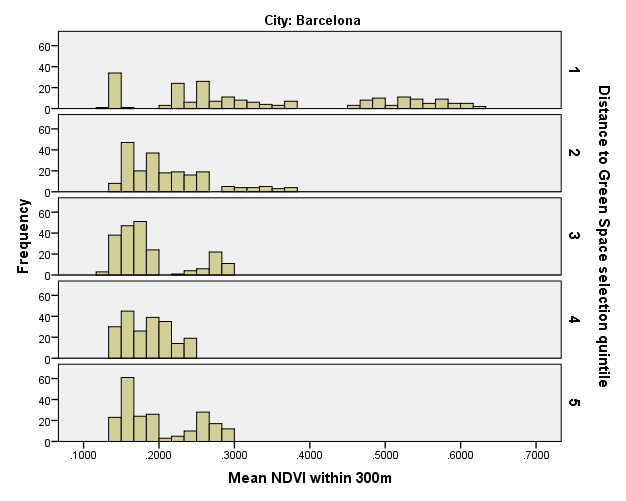


Figure S1a Distribution of mean NDVI within 300m by Neighbourhood selection distance to green space quintile (1 = lowest mean distance to green space, 5 = highest mean distance to green space) - Barcelona


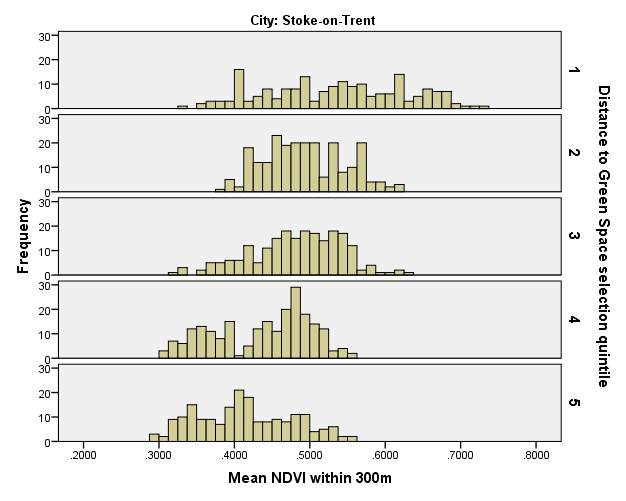
Figure S1b Distribution of mean NDVI within 300m by Neighbourhood selection distance to green space quintile (1 = lowest mean distance to green space, 5 = highest mean distance to green space) – Stoke-on-Trent


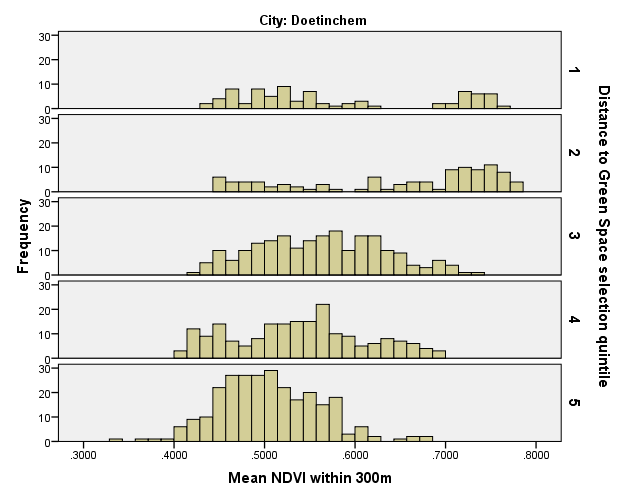
Figure S1c Distribution of mean NDVI within 300m by Neighbourhood selection distance to green space quintile (1 = lowest mean distance to green space, 5 = highest mean distance to green space) – Doetinchem


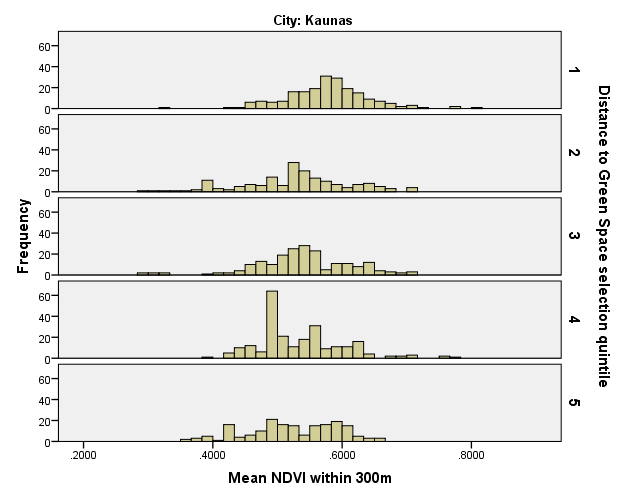
Figure S1d Distribution of mean NDVI within 300m by Neighbourhood selection distance to green space quintile (1 = lowest mean distance to green space, 5 = highest mean distance to green space) – Kaunas
